# ANNUAL WELLNESS VISITS (AWV) FOR RESIDENTS IN LOW-INCOME SENIOR HOUSING

**DOI:** 10.1093/geroni/igad104.0600

**Published:** 2023-12-21

**Authors:** Barbara Resnick, Jennifer Klinedinst, Nicole Brandt, Sarah Holmes

**Affiliations:** University of Maryland, Baltimore, Maryland, United States; University of Maryland School of Nursing, Baltimore, Maryland, United States; University of Maryland, Baltimore, Baltimore, Maryland, United States; University of Maryland School of Nursing, Baltimore, Maryland, United States

## Abstract

Despite the known benefits, less than one-fifth of all eligible Medicare patients receive an Annual Wellness Visit (AWV). Consequently, we brought the AWV to the older residents in low-income senior housing. Across all four communities the residents are invited to receive an AWV during their birthday month via an individual invitation. Invitations are sent to the facility manager who places them in the residents’ mailboxes. As per Medicare requirements, the Annual Wellness Visit includes such things as medical and family history, review of providers and/or suppliers, height, weight, blood pressure monitoring, medication review, evaluations of health behaviors, cognition, immunizations, mood, safety, and health screenings. At the end of the review the resident is provided with a set of health goals for the year and a small token birthday gift from the team. Approximately half of those who were invited each month came to the clinic for their Annual Wellness Visit. None of the residents to date who have had a Welcome to Medicare Visit were aware of the opportunity. Goals have focused on better adherence to health behaviors, particularly medication adherence, diet and physical activity, and updating immunizations. Most reported adherence to appropriate age-related screenings, and monitoring of cardiovascular disease and screening for and assessment of diabetes and hypercholesteremia. Lastly, we have been able to encourage the completion of the Maryland Orders for Life Sustaining Treatment among many of the participants who were previously unaware of this form and/or not willing to address it.

